# Improvement in Solubility–Permeability Interplay of Psoralens from *Brosimum gaudichaudii* Plant Extract upon Complexation with Hydroxypropyl-β-cyclodextrin

**DOI:** 10.3390/molecules27144580

**Published:** 2022-07-19

**Authors:** Rúbia Darc Machado, Júlio C. G. Silva, Luís A. D. Silva, Gerlon de A. R. Oliveira, Luciano M. Lião, Eliana M. Lima, Mariana C. de Morais, Edemilson C. da Conceição, Kênnia R. Rezende

**Affiliations:** 1 Laboratório de Biofarmácia e Farmacocinética (BioPk), Faculdade de Farmácia, Universidade Federal de Goiás, Goiânia 74605-170, GO, Brazil; drubia@egresso.ufg.br (R.D.M.); jcesagonzaga@gmail.com (J.C.G.S.); 2 Laboratório de Nanotecnologia Farmacêutica e Sistemas de Liberação de Fármacos (FarmaTec), Faculdade de Farmácia, Universidade Federal de Goiás, Goiânia 74605-170, GO, Brazil; luis.dantas@ufg.br (L.A.D.S.); emlima@ufg.br (E.M.L.); 3 Laboratório de Ressonância Magnética Nuclear (LabRMN), Instituto de Química, Universidade Federal de Goiás, Goiânia 74605-170, GO, Brazil; gerlonribeiro@gmail.com (G.d.A.R.O.); lucianoliao@ufg.br (L.M.L.); 4 Laboratório de PD&I de Bioprodutos, Faculdade de Farmácia, Universidade Federal de Goiás, Goiânia 74605-170, GO, Brazil; marianacmfarma@gmail.com (M.C.d.M.); edemilson_conceicao@ufg.br (E.C.d.C.)

**Keywords:** cyclodextrin, HP-β-CD, inclusion complexes, physical–chemical characterization, host–guest interaction, solubilization, ex vivo permeability, intestinal absorption, in vitro studies, furanocoumarins

## Abstract

Psoralen (PSO) and 5-methoxypsoralen (5-MOP) are widely used drugs in oral photochemotherapy against vitiligo and major bioactive components of root bark extract of *Brosimum gaudichaudii* Trécul (EBGT), previously standardized by LC-MS. However, the exceptionally low water solubility of these psoralens can cause incomplete and variable bioavailability limiting their applications and patient adherence to treatment. Therefore, the purpose of this work was to investigate the effects of 2-hydroxypropyl-β-cyclodextrin (HP-β-CD) inclusion complex on the solubility and jejunal permeability of PSO and 5-MOP from EBGT. Characterization of inclusion complexes were evaluated by current methods in nuclear magnetic resonance studies on aqueous solution, Fourier transform infrared spectroscopy, thermal analysis, and scanning electron microscopy in solid state. Ex vivo rat jejunal permeability was also investigated and compared for both pure psoralens and plant extract formulation over a wide HP-β-CD concentration range (2.5 to 70 mM). Phase solubility studies of the PSO- and 5-MOP-HP-β-CD inclusion complex showed 1:1 inclusion complex formation with small stability constants (K_c_ < 500 M^−1^). PSO and 5-MOP permeability rate decreased after adding HP-β-CD by 6- and 4-fold for pure standards and EBGT markers, respectively. Nevertheless, the complexation with HP-β-CD significantly improved solubility of PSO (until 10-fold) and 5-MOP (until 31-fold). As a result, the permeability drop could be overcome by solubility augmentation, implying that the HP-β-CD inclusion complexes with PSO, 5-MOP, or EBGT can be a valuable tool for designing and developing novel oral drug product formulation containing these psoralens for the treatment of vitiligo.

## 1. Introduction

Psoralen (PSO) and 5-methoxypsoralen (5-MOP) are linear furocoumarins, also known psoralens, commonly employed in oral photochemotherapy against different skin disorders, such as psoriasis and vitiligo. Mostly, 5-MOP shows less acute side effects and a slightly higher tolerance in patients in comparison to other photosensitizers [1]. In this context, *Brosimum gaudichaudii* Trécul (Moraceae) has attracted a great deal of attention due to large accumulation of these psoralens in its roots. Other chemically related coumarins previously described for *B. gaudichaudii* include gaudichaudine, xanthyletin, luvangetin, (+)-(2′S,3′R)-1′-hydroxy-marmesin, marmesin, 1′,2′-dehydromarmesin, 8-methoxymarmesin, and marmesinin [2,3,4]. Furthermore, this species has long been used as folk medicine in vitiligo’s treatment [5,6,7]. Recently, Quintão et al. (2019) brought additional evidence on eliciting properties of *B gaudichaudii* inducing melanocyte migration and skin pigmentation in vitro [8]. However, very low water solubility of psoralens can cause incomplete and variable bioavailability, limiting oral administration [9,10,11,12,13,14].

During both drug development and quality control process, complexation of poorly water-soluble phytochemicals with parent-cyclodextrin (CD) and their derivatives have emerged as a valuable tool to optimize pharmaceutical properties, such as solubility/permeability, stability, and toxicity and thus enhance drug delivery systems and quality control issues [15]. Thus, improving physicochemical properties of phytochemicals can enhance their biological activities, sensory properties, and effectively improve patient adherence to treatment. Particularly, CD-inclusion complexes with phenolic plant compounds can enhance the water solubility and mask the bitterness of catechins [16].

Currently, there are different techniques to obtain and characterize cyclodextrin inclusion complexes. The most used in the last few years include co-precipitation, kneading, super critical carbon dioxide, spray drying, and freeze drying, among others, with their pros and cons, as elsewhere discussed [17]. Characterization methods involve the use of several analytical techniques, mostly depending on the physical nature of the complex (liquid or solid state). Typically, data-based evidence is shown by physical or chemical property variations of the guest molecule due to the host-guest interactions. For solid state, the major analytical techniques are thermogravimetric analysis (TGA) and differential scanning calorimetry (DSC), together with scanning electron microscopy (SEM), Fourier-transform infrared spectroscopy (FTIR), powder X-ray diffraction (PXRD), and Raman spectroscopy [18]. Some of them, such as powder X-ray diffraction (PXRD), are usually not suitable for providing structural information when putative complexes are as an amorphous powder [17]. 

Psoralen inclusion complexes with heptakis-(2,3,6-tri-O-methyl)-β-cyclodextrin (TRIMEB), β-CD, heptakis-(2,6-di-O-methyl)-β-cyclodextrin (DM-β-CD), and hepatakis-(2,3,6-tri-O-methyl)-β-cyclodextrin (TM-β-CD) in solid state have been investigated and confirmed by spectroscopic and/or thermal analyses [19,20,21]. At present, oral administration of methylated β-cyclodextrin is limited by its potential cytotoxic properties [22,23,24]. On the other hand, drug complex formation with HP-β-CD is most favorable both when increasing solubility and decreasing toxicity. Thus, HP-β-CD has often been used as an alternative to parent β-CD, and its methylated derivatives, except the HP-β-CD impact on intestinal permeability, is still poorly understood [25].

In this sense, drug/CD complexes formation can increase drug solubility, but only the free guest molecules in the equilibrium with the complex itself are able to permeate biological membranes. It is well-known that cyclodextrins can modulate, improve, or hamper the permeability through biological barriers. Improved permeability can be seen for hydrophobic drugs (BCS class II) as the water-soluble inclusion complex may eliminate the unstirred water layer (UWL), facilitating drug transport to membrane surface. Instead, permeability decreases in an excess amount of cyclodextrin in oral dosage forms [23,26,27,28]. Therefore, to maximize drug absorption, the CD amount added into pharmaceutical preparation needs to be carefully evaluated.

From our previous studies [2], PSO and 5-MOP showed limited aqueous solubility, both being classified as class II drugs, according to the Biopharmaceutical Classification System (BCS), suggesting that their absorption is markedly affected by its aqueous solubility. Therefore, these psoralens are very promising drugs for cyclodextrin complexation. The purpose of this work was to evaluate and optimize their solubility/permeability by inclusion with HP-β-cyclodextrin, characterizing host-guest interactions between HP-β-CD, PSO, and 5-MOP from *B. gaudichaudii*.

## 2. Results and Discussion

### 2.1. Phase Solubility Studies

**Figure 1:**
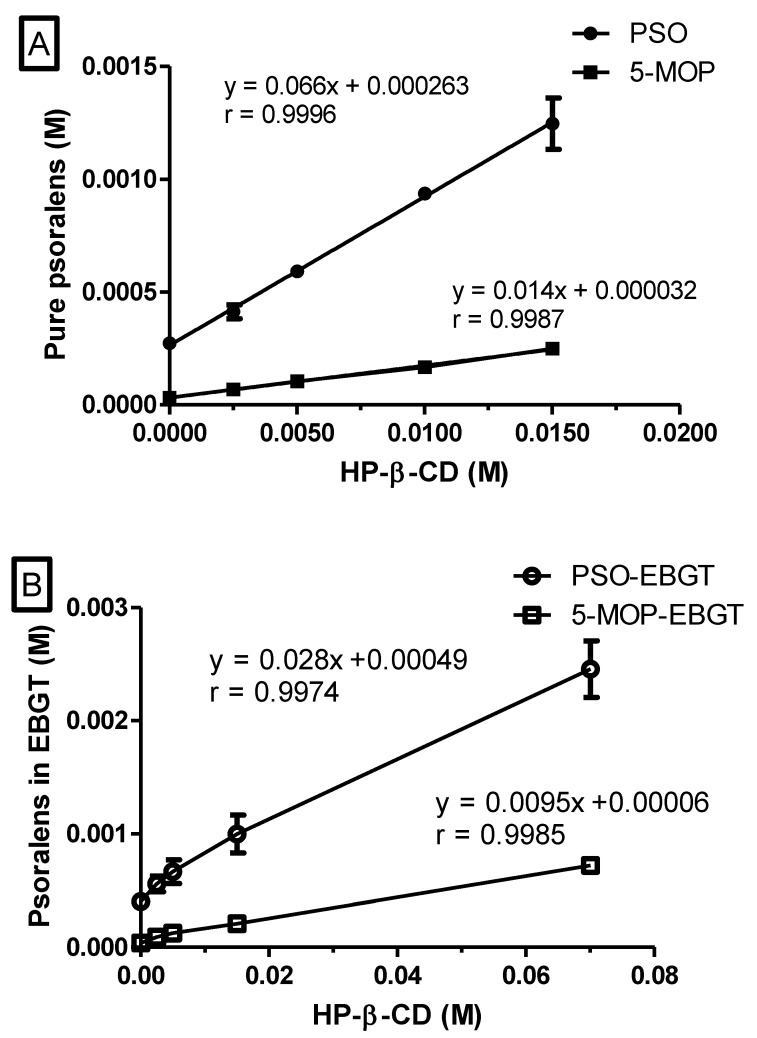
Solubility of psoralens standards (**A**) and psoralens in EBGT (**B**) as a function of HP-β-CD concentration in KRB buffer solutions (pH 7.4; 37 °C). Data shown as mean ± SD (error bars smaller than symbols); *n* = 3.

The phase solubility diagram obtained for the PSO- and 5-MOP-HP-β-CD inclusion complex is shown in Figure 1A. The intrinsic solubility (S_0_) was found to be 0.273 ± 0.007 mM (*n* = 3) and 0.032 ± 0.001 mM (*n* = 3), respectively. The solubility of these psoralens linearly increased (r > 0.999) as a function of HP-β-CD concentration, and slopes were less than one, so the profile of the diagram corresponds to A_L_ type, indicating 1:1 molecular complex formation. The K_c_ calculated for PSO- and 5-MOP-HP-β-CD were 259.2 ± 29.1 M^−1^ (*n* = 3) and 449.9 ± 16.6 M^−1^ (*n* = 3), respectively, exhibiting a very weak interaction profile and values within the range previously stated (50–5000 M^−1^) [29].

For the EBGT markers, the phase solubility diagram obtained for PSO- and 5-MOP-HP-β-CD inclusion complex was also A_L_ type, indicating 1:1 molecular complex formation, according to the Higuchi-Connor classification system (Figure 1B). However, The K_c_ was about 3.6- and 1.8-fold lower when compared to the respective pure compounds. The K_c_ values for PSO and 5-MOP in EBGT were 72.5 ± 0.9 M^−1^ (*n* = 3) and 251.2 ± 1.4 M^−1^ (*n* = 3), respectively. Such values indicate that, under in vivo conditions, these complexes will readily dissociate into separate components [25,29].

The intrinsic solubility (S_0_) of PSO and 5-MOP in EBGT (9.0 mg/mL) was found to be 0.40 ± 0.02 mM (*n* = 3) and 0.038 ± 0.002 mM (*n* = 3), respectively. Thus, the experimental value of intrinsic psoralens solubility (S_0_) was also affected by the plant matrix and demonstrates positive deviation of the extrapolated value from the phase-solubility line. As demonstrated by Kurkov, Ukhatskaya, and Loftsson (2011) [30], this behavior is common among poorly soluble drugs. 

Such intrinsic solubility uncertainty can yield great fluctuations in the stability constant values obtained from phase-solubility diagrams. Thus, complexation efficiency (CE) is taken as a better parameter for comparing solubilization effects of HP-β-CD over psoralens at different environments, since it is independent of intrinsic solubility and the intercept [31].

CE values of PSO/ and 5-MOP/HP-β-CD complexes decreased from 0.071 ± 0.008 (*n* = 3) to 0.029 ± 0.000 (*n* = 3) and from 0.014 ± 0.001 *(n* = 3) to 0.010 ± 0.001 (*n* = 6), respectively, in EBGT. This means that around 7.1% of the HP-β-CD molecules in the solution are forming water soluble complexes with pure PSO, while only about 2.9% of cyclodextrin is forming complexes with PSO in EBGT [25,31,32]. For 5-MOP, there was no important change in the complexation efficiencies as a function of plant matrix. Taken together, results suggest a competitive role played by other phytochemicals in plant matrix, mainly with PSO, by cyclodextrin cavity.

To our knowledge, there is no previous report regarding the encapsulation of 5-MOP and *B. gaudichaudii* extract in HP-β-CD. Regarding the EBGT, it is worthwhile to consider that the herbal extracts are multicomponent mixtures where hundreds of compounds can coexist, albeit coming from a single plant. Thus, providing definitive experimental evidence of the inclusion complex formation of one specific phytochemical in their plant matrix with cyclodextrins is a rather complicated task, especially in solid state. Although the determination of phase-solubility profiles does not indicate whether EBGT markers form an inclusion complex with HP-β-CD, it can be a source of valuable information on how the HP-β-CD influences the psoralens solubility in plant matrix. 

Additionally, phase-solubility profile indicated that the highest enhancement in the solubility was seen for 5-MOP-HP-β-CD inclusion complexes, followed by 5-MOP/HP-β-CD in plant extract. Next, it was seen for PSO complex as a pure compound and then for PSO-HP-β-CD in plant extract.

### 2.2. Nuclear Magnetic Resonance Characterization

**Figure 2:**
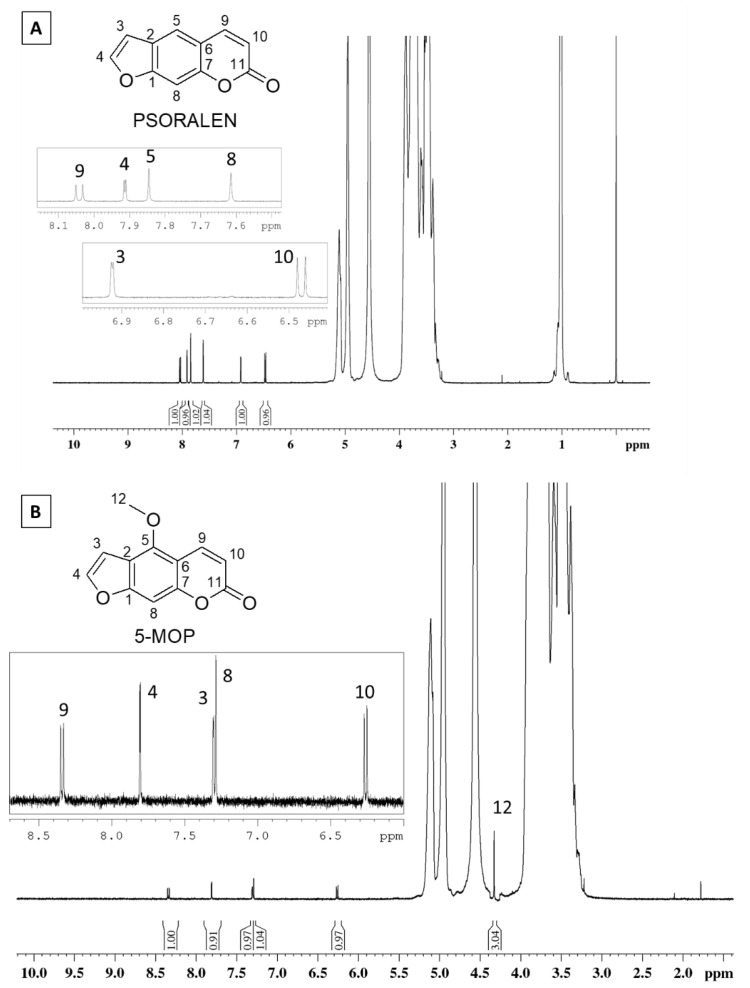
^1^H NMR spectrum of the (**A**) PSO- and (**B**) 5-MOP-HP-β-CD inclusion complex (HP-β-CD 15 Mm, D_2_O, 500 MHz).

Most CD applications take place in solution, so NMR spectra are especially important once allowing the characterization of complex formation to be in this state. The furanocoumarins signals observed in the ^1^H NMR spectra are shown in Figure 2. Their signals were less intense than those from HP-β-CD (*δ* 3.0 to 5.5) due to the lower concentration of the coumarins. 

**Figure 3:**
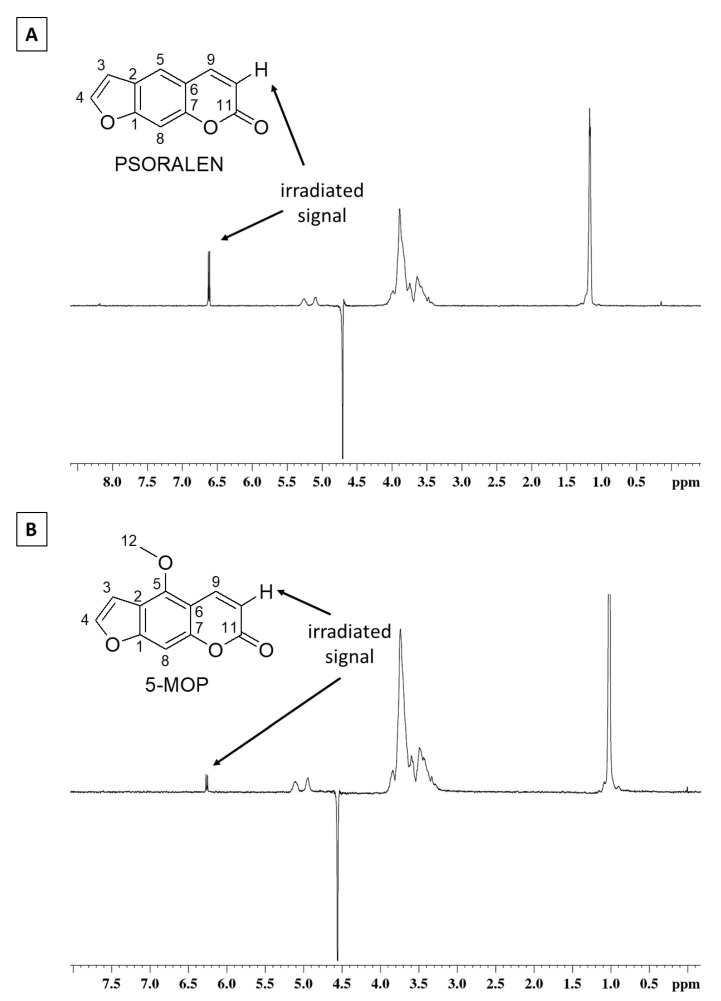
NOESY experiments for PSO (**A**) and 5-MOP (**B**) solutions. H-10 signals were irradiated, and polarization transfers to HP-β-CD were observed.

Selective irradiation of H-10 hydrogen in 5-MOP and PSO (Figure 3) clearly shows an increase of the HP-β-CD (*δ* 3.0 to 4.0) signals due to the polarization transfer. This kind of interaction, typical of nuclei with spatial proximity, is strong evidence of complexation between furanocoumarins and HP-β-CD.

**Figure 4:**
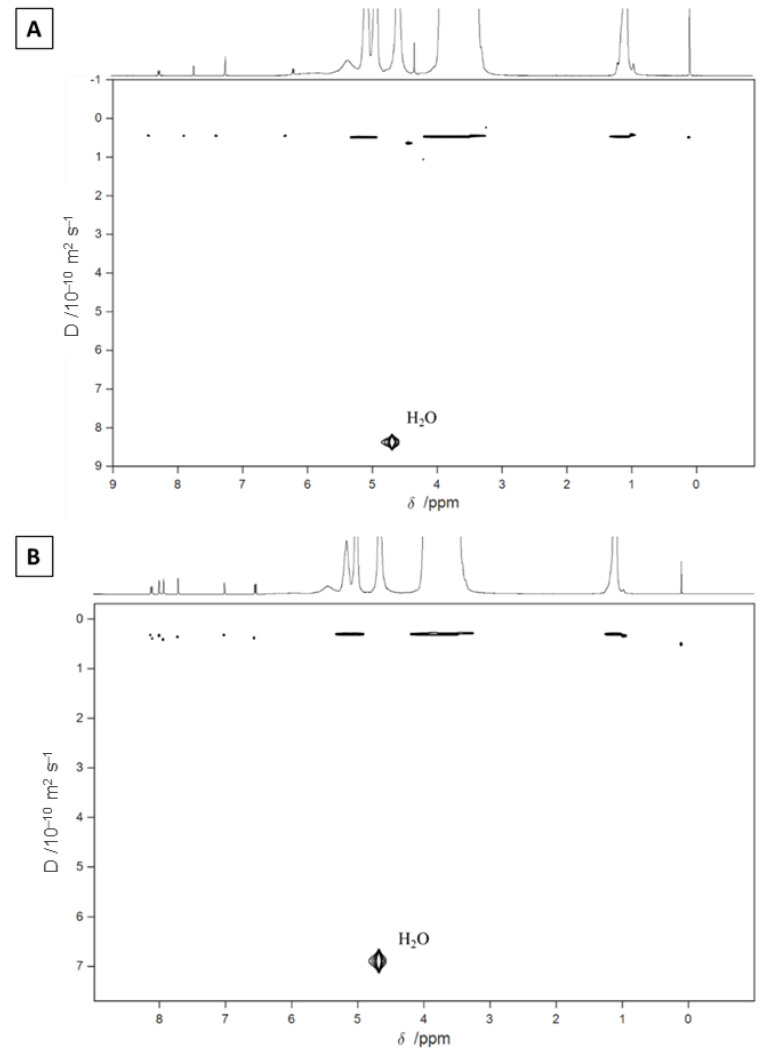
^1^H DOSY experiments for 5-MOP/HP-β-CD (**A**) and PSO/HP-β-CD (**B**) (500 MHz, D_2_O).

The complexation between the furanocoumarins and HP-β-CD was also corroborated by Diffusion Ordered Spectroscopy (DOSY). In this experiment, chemical shifts and diffusion coefficients were presented in two orthogonal directions that effectively separate out the NMR signals according to the diffusion coefficients of the components [33]. Furanocoumarins and HP-β-CD have a large difference in molecular weight, so they have different diffusion coefficients. However, in DOSY experiments (Figure 4), only one component signal was observed (0.45 × 10^−10^ m^2^ s^−1^ of diffusion), in addition to the D_2_O signal, demonstrating the host-guest complex between the furanocoumarins and HP-β-CD. In order to confirm the joint diffusion demonstrating complexation of these compounds, the values of the total diffusion-encoding pulse duration and the diffusion delays were varied (p30 = 1 to 2 ms; d20 = 50 to 100 ms), and no separation between furanocoumarins and HP-β-CD was observed. 

### 2.3. Characterization of the PSO- and 5-MOP-HP-β-CD Inclusion Complex in Solid State

#### 2.3.1. Thermogravimetry (TG)

**Figure 5:**
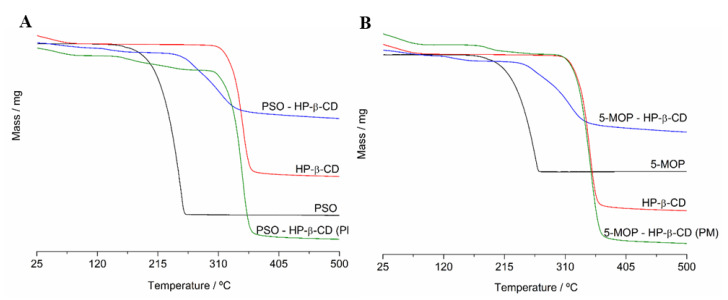
(**A**) TGA curves of PSO, HP-β-CD, physical mixture (PM), and PSO-HP-β-CD inclusion complex (1:1). (**B**) TGA curves of 5-MOP, HP-β-CD, physical mixture (PM), and 5-MOP-HP-β-CD inclusion complex (1:1).

The TGA curves of PSO, 5-MOP, HP-β-CD, PSO-HP-β-CD, or 5-MOP-HP-β-CD inclusion complex (1:1) and physical mixtures are shown in Figure 5. The TGA curves of HP-β-CD show a typical decomposition temperature at 343.0 °C (onset temperature = 331.7 °C), with a mass change of 88.95%. The overall mass change was 93.6%. Dehydration of HP-β-CD (loss of water molecules from the cavity) was observed in a range of 36.5 to 65.0 °C. 

The TGA curve of free PSO revealed a unique representative decomposition peak at the onset temperature of 223.2 °C (weight loss of 99.5%), while the 5-MOP decomposition peak occurred at the onset temperature of 231.9 °C (weight loss of 98.0%). 

The physical mixture exhibited a similar three-step weight loss for PSO (30–65 °C, 223.2 °C, and 328.6 °C) and 5-MOP (30–65 °C, 231.9 °C, and 330.6 °C), corresponding to evaporation of water from HP-β-CD, drug decomposition, and HP-β-CD decomposition, respectively. This indicated that a physical mixture was not able to incorporate psoralens inside the nonpolar cavity of HP-β-CD.

On the other hand, the TGA curves of the PSO and 5-MOP in inclusion complex showed a higher degradation temperature compared to single components: shift from 223.18 to 271.50 °C for PSO-HP-β-CD and from 231.89 to 275.78 °C for 5-MOP-HP-β-CD inclusion complex. In general, the degradation of the guest molecule takes place at higher temperatures when complexation occurs, since the drug is protected by cyclodextrin [34].

#### 2.3.2. Differential Scanning Calorimetry (DSC)

**Figure 6:**
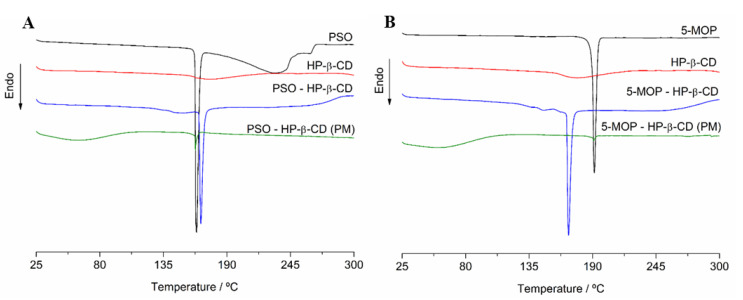
(**A**) Differential scanning calorimetry thermograms of PSO, HP-β-CD, physical mixture, and PSO-HP-β-CD inclusion complex (1:1). (**B**) Differential scanning calorimetry thermograms of 5-MOP, HP-β-CD, physical mixture (PM), and 5-MOP-HP-β-CD inclusion complex (1:1).

In particular, DSC is the most widely used technique for the evaluation of inclusion compound formation in solid-state [17]. Figure 6 shows DSC thermal curves of both pure psoralens (PSO, 5-MOP), guest molecule (HP-β-CD), the putative (1:1) inclusion complexes (PSO-HP-β-CD or 5-MOP-HP-β-CD), and their physical mixtures. 

For the guest molecule spectra (HP-β-CD), a small endothermic state transition effect appears at 176.0 °C with an exceptionally low mass loss. The typical broad endothermic peak of HP-β-CD (around 341 °C), which is attributed to the decomposition of the HP-β-CD, could not be observed in the temperature range herein evaluated. 

Free PSO and 5-MOP showed a well-defined endothermic peak in 163.8 °C and 190 °C, respectively, which corresponds to their melting points of crystal forms.

DSC thermal curve of the putative inclusion complexes (PSO-HP-β-CD or 5-MOP-HP-β-CD) showed temperature profiles differing from both host molecules, guest molecules, and their physical mixture, which still exhibited the endothermic peak of the drugs at the same melting points of crystal forms. 

Notably, the spectra of PSO-HP-β-CD and 5-MOP-HP-β-CD did not show PSO or 5-MOP peaks correspondent to pure compounds, respectively. Instead, it showed a shift of melting peak of PSO (163.81 to 168.28 °C) and 5-MOP (190.02 to 168.11 °C), which is generally taken as evidence of complexation and/or amorphization of the inclusion complex in the solid state. 

The disappearance of the melting peak of the drug or the shift and/or the flattening of the DSC profile in the melting point region of the crystalline drug is often taken as robust evidence of inclusion complex formation [35]. Additionally, it can also be seen as indicative of a residual presence of the drug with a partial reduction in the crystallinity, suggesting amorphization and/or incomplete inclusion in the guest molecule. Both findings are well documented by an overwhelming number of publications covered by a recent review article [17]. 

During drug development, the inclusion complex encapsulation ratio can be measured by a joint analytical approach, together with further studies, as reported for apigenin- HP-β-CD with -chitosan ternary complex formulation [36], including additional analysis, such as XRD evaluation and formulation dissolution. Here, the formation of the inclusion complex of psoralens and HP-β-CD were investigated by four different methods in solid state: Differential Scanning Calorimetry (DSC); Thermogravimetry (TG); Fourier transform infrared spectroscopy (FTIR), and Scanning electron microscopy (SEM), showing findings corroborating with the evidence of inclusion complex formation (see details on the following sections). Although evidenced in our earlier study [2], the EBGT showed amorphous properties, seen by the absence of peaks in X-ray diffraction spectra after the freeze-drying process, so that XRD would not be very suitable to provide useful structural information. It is worth remarking that the primary aim of our work was to investigate HP-β-CD complex formation with PSO and 5-MOP from *B. gaudichaudii* impacting on solubility and permeability through GT mucosa. When practical, such remarkable analytical progress may allow a more comprehensive evaluation of the prepared inclusion complex in order to prevent loss of formulation quality control, e.g., drug recrystallization.

#### 2.3.3. Fourier Transform Infrared Spectroscopy (FTIR)

**Figure 7:**
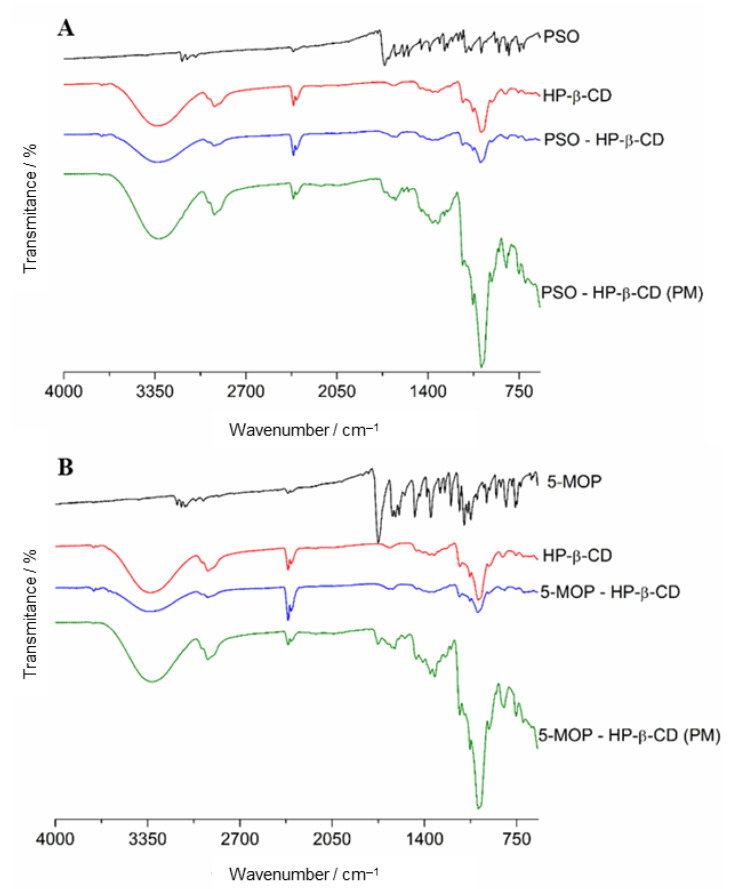
(**A**) ATR-FTIR spectra of PSO, HP-β-CD, physical mixture (PM), and PSO-HP-β-CD inclusion complex (1:1). (**B**) ATR-FTIR spectra of 5-MOP, HP-β-CD, physical mixture (PM), and 5-MOP-HP-β-CD inclusion complex (1:1).

The FTIR spectrum of PSO, 5-MOP, HP-β-CD, PSO-HP-β-CD, or 5-MOP-HP-β-CD inclusion complex (1:1) and physical mixtures are shown in Figure 7. The FTIR spectral analysis of pure PSO showed principal peaks at wavenumbers 3155, 3120, and 3061 (aromatic C–H stretch); 1711 (C=O stretching vibration of unsaturated lactones); 1448; 1573 (C=C of ring); 1226 (C–O–C ether group); 1092–1130 (C–O stretching vibration); 1020 (O–C–C band); and 748 (out of plane C–H bend). 

For 5-MOP, the values of absorption band integration were noted at wavenumbers 3074 and 3086 (aromatic C–H stretch); 2847 and 2958 (C–H stretch from methyl group); 1726 (C=O stretching vibration of unsaturated lactones); 1605; 1468 (C=C of ring); 1257 (asymmetric C–O–C stretch of aryl ethers); 1030–1121 (C–O stretching vibration); 1153 (O–C–C band); and 760 (out of plane C–H bend). 

The FTIR spectra of HP-β-CD exhibited intense absorption peak at 3326.6 cm^−1^, related to the O–H stretching vibration. Other HP-β-CD characteristic bands were shown in the regions of 2925.5 cm^−1^ (C–H bond stretching vibrations), 1151 cm^−1^ (C−H bond stretching vibrations), and 1080 cm^−1^ (C–O bond stretching vibrations), as reported by Medarević et al. [37]. 

The spectrum of physical mixtures of these psoralens with HP-β-CD was almost a simple overlapping of pure components spectra. On the other hand, the spectrum of the inclusion complex of both PSO and 5-MOP was identical to that of pure HP-β-CD, suggesting that the drugs are completely inside the truncated-cone formed by cyclodextrin.

#### 2.3.4. Scanning Electron Microscopy (SEM)

**Figure 8:**
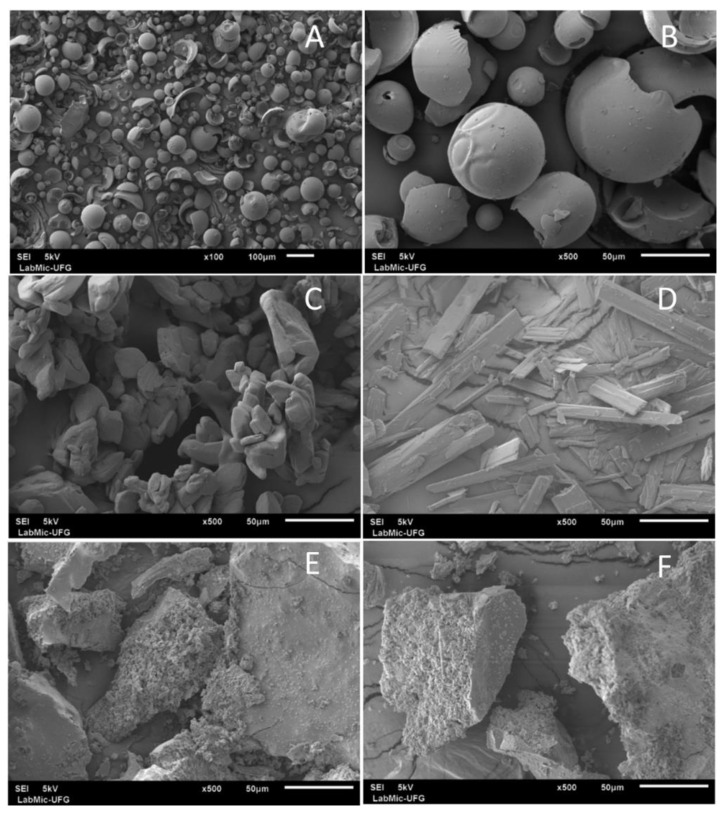
Scanning electron photomicrographs of (**A**) HP-β-CD; (**B**) HP-β-CD; (**C**) PSO; (**D**) 5-MOP; (**E**) PSO-HP-β-CD inclusion complex (1:1); (**F**) 5-MOP-HP-β-CD inclusion complex (1:1). 100× magnification (**A**) and 500× magnification (**B**–**F**).

Representative SEM images of PSO, 5-MOP, HP-β-CD, PSO-, and 5-MOP-HP-β-CD inclusion complex (1:1) are shown in Figure 8. SEM analysis of HP-β-CD shows smooth and spherical shaped particles of different sizes. In contrast, pure psoralen was characterized by the presence of uneven and broken particles, while pure 5-MOP was characterized by the presence of crystals, showing a rectangular shape. The typical morphological characteristics of pure psoralens were changed in the inclusion complex that shows different sizes, with a predominantly smooth surface and with a porous inside for both PSO- and 5-MOP-HP-β-CD inclusion complexes. These results suggested the formation of complexes of psoralens with HP-β-CD in the solid-state. Our findings are in agreement with observations from formulation of genistein-HP-β-C-Poloxamer 188 ternary inclusion complex [38].

### 2.4. Ex Vivo Oral Permeability Assessment 

Permeability of psoralens was evaluated by using increasing concentrations of HP-β-CD by using rat jejunal ex vivo assay, previously validated showing excellent correlation to intestinal absorption in humans [39].

The effects of HP-β-CD on the viability of intestinal tissue segments mounted in the MTS-Snapwell system was evaluated during incubation time (120 min) at 37 °C. For this purpose, TEER values in the presence and absence of 20% (*w*/*w*) HP-β-CD were compared. Values of the TEER, after incubation, containing this cyclodextrin derivative (29 ± 7 Ω·cm^2^, *n* = 16), did not differ (*p* = 0.99) from the values for control KRB buffer solution (32 ± 9 Ω·cm^2^, *n* = 16). These results clearly suggest that the HP-β-CD, even at high concentration, does not affect the viability of jejunal tissue.

To permeate the enterocytes cells, most lipophilic drugs need to overcome the unstirred water layer (UWL) adjacent to mucosal epithelium. Depending on thickness, UWL becomes the main barrier, i.e., permeation becomes diffusion controlled. According to Loftsson [40], under unstirred in vitro condition, the UWL can be significantly thicker (until 1000 µm or more) than in a typical in vivo environment (up to about 100 µm). Therefore, it is important to monitor the relative resistance of the drug flux through UWL. This resistance can be examined through a comparison of the permeation on stagnated and stirring conditions [28].

In order to evaluate UWL contribution on permeability of psoralens, the assay was carried out on MTS-Snapwell plate with (60 rpm) and without stirring (0 rpm) during 120 min. Results showed no statistical difference on P_app_ of PSO (21.38 ± 3.81 vs. 20.49 ± 2.82 × 10^−6^ cm/s; *n* = 6; *p* = 0.63) or 5-MOP (11.16 ± 3.28 vs. 7.80 ± 1.91 × 10^−6^ cm/s; *n* = 6; *p* = 0.052), respectively, suggesting that membrane plays the main role in the permeation of psoralens, i.e., the UWL barrier is negligible. In general, hydrophilic CDs, such as HP-β-CD, do not enhance drug delivery through membranes if no UWL is present [41].

**Figure 9:**
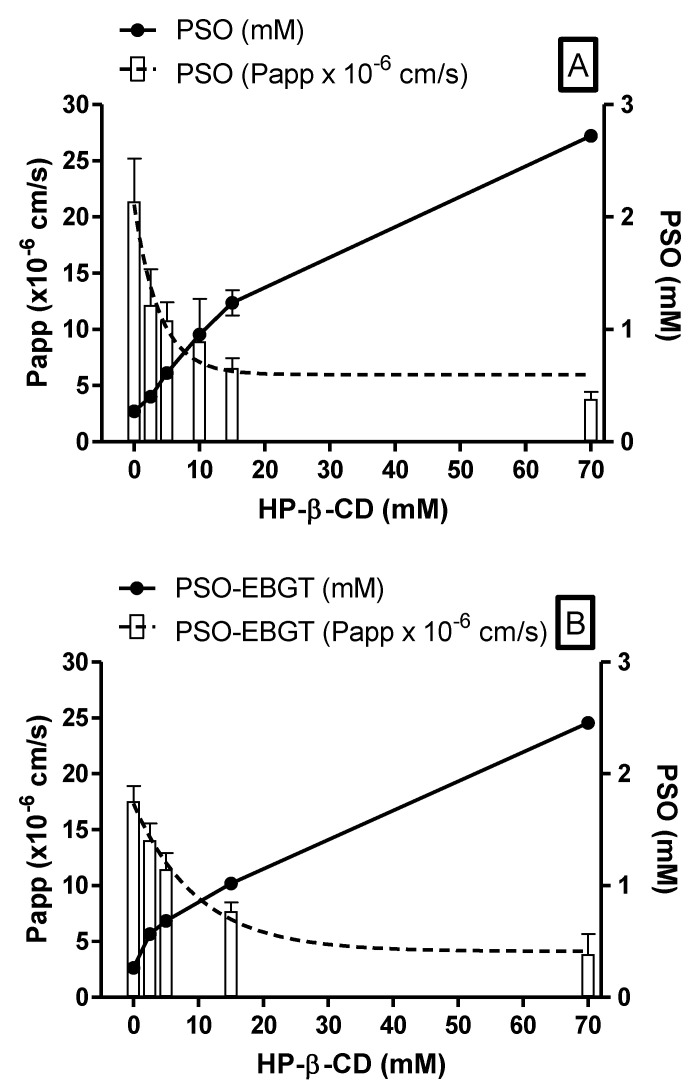
Ex vivo P_app_ and solubility of PSO standard (**A**) and PSO EBGT marker (**B**) as a function of increasing HP-β-CD concentration. P_app_ shown as mean ± SD (*n* = 6) and expressed as ×10^–6^ cm/s; solubility shown as mean ± SD (*n* = 3) and expressed as mM.

Regarding solubility of psoralens, it was significantly improved by HP-β-CD. By adding 70 mM HP-β-CD (*w*/*w*), PSO aqueous solubility, as pure compound or in plant extract, was improved from 0.27 ± 0.007 to 2.72 ± 0.03 mM and 0.40 ± 0.02 to 2.46 ± 0.03 mM, respectively, corresponding to a solubility increase of 10- and 6-fold (Figure 9A,B). Nevertheless, it is worth mentioning that, for the pure compound, a plateau was achieved upon completion of existing PSO (2.5 mg) in the HP-β-CD complexation media (5.0 mL) at 70 mM (Figure 9A). It is assumed that if more PSO is added by extrapolation, the expected value should be approximately 4.88 mM.

In contrast, PSO permeability as a pure compound and in plant extract decreased exponentially from 21.38 ± 3.81 and 17.56 ± 1.35 × 10^−6^ cm/s in KRB solution to 3.82 ± 0.62 and 3.86 ± 1.81 × 10^−6^ cm/s in HP-β-CD 70 mM, respectively, i.e., about 5.5-fold of the permeability rate (Figure 9A,B).

**Figure 10:**
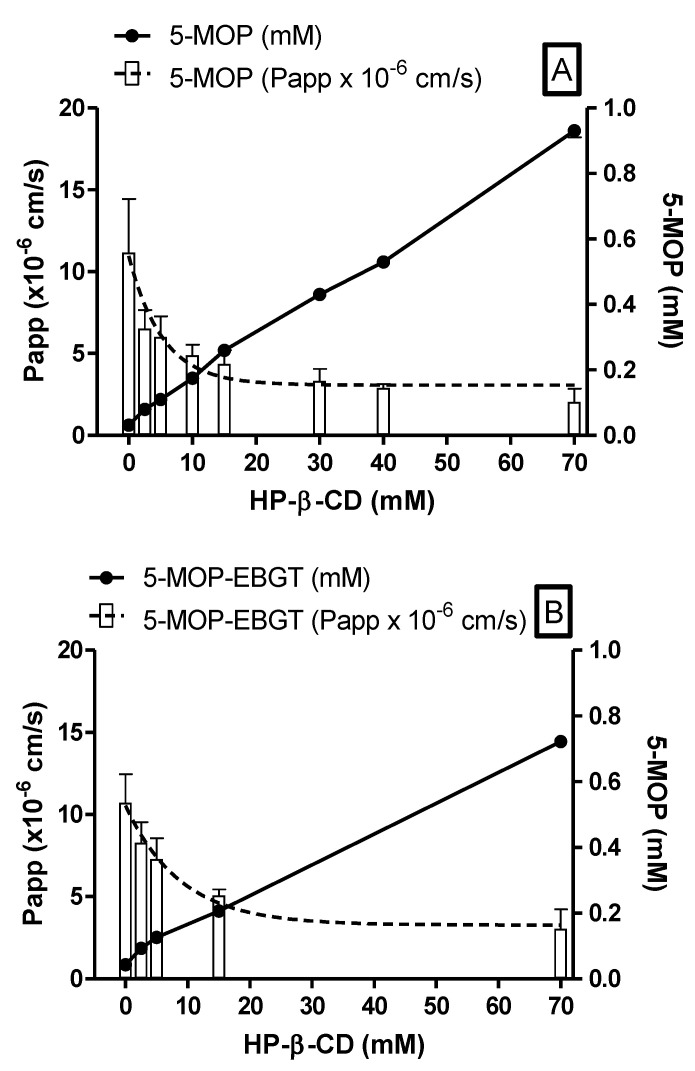
Ex vivo P_app_ and solubility of 5-MOP standard (**A**) and 5-MOP EBGT marker (**B**) as a function of increasing HP-β-CD concentration. P_app_ shown as mean ± SD (*n* = 6) and expressed as ×10^–6^ cm/s; solubility shown as mean ± SD (*n* = 3) and expressed as mM.

Aqueous solubility of 5-MOP, which was nearly insoluble as a free compound (uncomplexed), had drastically increased after its inclusion on HP-β-CD cavity. Aqueous solubility of 5-MOP as a pure compound and in plant extract was improved from 0.03 ± 0.001 to 0.93 ± 0.007 mM and 0.04 ± 0.002 to 0.72 ± 0.04 mM, respectively, by adding 70 mM HP-β-CD (*w*/*w*), corresponding to a solubility increase of 31- and 18-fold (Figure 10A,B). 

Ex vivo permeability studies of 5-MOP also showed an exponential decrease when increasing HP-β-CD concentration. Permeability of 5-MOP-HP-β-CD inclusion complex, both as a pure compound or as plant extract decreased from 11.16 ± 3.28 and 10.73 ± 1.73 × 10^−6^ cm/s in KRB buffer to 2.05 ± 0.81 and 3.04 ± 1.19 × 10^−6^ cm/s in HP-β-CD 70 mM solution, respectively (Figure 10A,B). 

These observations are in agreement with findings from permeability studies of progesterone (BCS class II drug), where cyclodextrins may exponentially reduce permeability even for lipophilic compounds [28], indicating the existence of solubility-permeability interplay/tradeoff phenomenon. The exact mechanism by which this phenomenon occurs is not fully understood. For EBGT markers, P_app_ decreases slower than pure compounds. A higher permeability rate for EBGT was probably due to a higher free drug amount available for membrane permeation as a result of its higher solubility and lower affinity for HP-β-CD, when compared with pure compounds.

Introducing pharmaceutical formulations into the field of natural medicine is useful and promising once it offers effective and reliable delivery of medicinal phytochemicals [42]. Although various solubility-enabling formulations can be applied to the development of oral dosage forms, most of the pharmaceutical excipients (e.g., cosolvents, cyclodextrins, surfactants, hydrotropes, etc.) may affect the permeability in opposite directions [43,44]. However, its drug permeability impact depends on the amount of excipient used. Then, to maximize psoralens absorption, the CD amount added into pharmaceutical preparation needs to be carefully evaluated, aiming at the desired drug bioavailability intended for therapeutic benefits.

The solubility-permeability trade-off phenomenon, as well as defining their interplay, is an emerging challenge in today’s drug development. The primary approach to overcome this issue is using the minimal excipient amounts sufficient to dissolve the drug dose throughout the GIT. In other words, when the intrinsic permeability of the drug is very high, it may be useful to waste some permeability to gain solubility [43,45]. 

In this context, permeability drawback of the obtained psoralens complexes could be overcome by their improved solubility, meaning that comparable plasma concentrations can be reached at a lower dose than uncomplexed ones, as also evidenced by the closely resembling AUC of 5-MOP (30,977 ± 4742 vs. 22,645 ± 8630 ng/mL·min, *n* = 3) herein obtained in preliminary in vivo studies in rats at about a 10-fold lower dose of EBGT in HP-β-CD 70 mM solution (1.5 g/kg vs. 0.12 mg/kg) (*data not shown*). For comparison purposes, P_app_ found for metoprolol (0.58 mM), a BCS-marker used as low/high permeability class boundary drug, was 6.21 ± 2.63 × 10^−6^ cm/s (*n* = 41) [2]. Hence, psoralens complexed with HP-β-CD in the concentration range of 2.5 to 15.0 mM, in either the EBGT’s plant matrix or as pure compound, could be considered as high to moderate permeability compounds.

## 3. Materials and Methods

### 3.1. Solvents and Chemicals

Analytical grade 5-methoxypsoralen (≥99%) and psoralen (≥99%) were purchased from Sigma-Aldrich (St. Louis, MO, USA). Chemicals used for the Krebs-Ringer Bicarbonate buffers (KRB), NaCl, KCl, CaCl_2_.2H_2_O, NaH_2_PO_4_, MgSO_4_.7H_2_O, and NaHCO_3_, were purchased from Sigma-Aldrich (St. Louis, MO, USA) as well. HPLC-grade methanol, acetonitrile, and trifluoroacetic acid were obtained from Merck (Darmstadt, Germany). HP-β-CD (Cavitron W7 HP5 Pharma HPB^®^) was kindly provided by Ashland. 

### 3.2. Plant Material

Root bark extract of *B. gaudichaudii* Trécul (EBGT) was provided by Dr. Edemilson Cardoso da Conceição (Laboratório de PD&I de Bioprodutos, Federal University of Goiás, Goiânia, Brazil). Briefly, *B. gaudichaudii* root bark (voucher specimen number 45517) was cleaned, dried, crushed, ground in a mill, and percolated in 55% ethanol. The solution was evaporated to yield a solid content higher than 75% (*w*/*w*) [46], which was freeze-dried (−20 °C, 905 mTOR; Genesis SQ Super X-70, SP Scientifc^®^, Warminster, PA, USA). Plant extract was frozen just once and immediately before freeze-drying in the absence of cryoprotectant, as HP-β-CD itself is considered to have such properties. The PSO and 5-MOP content (%, *w*/*w*) in EBGT was determined by HPLC-DAD following a validated method described in the *HPLC-DAD method* section.

### 3.3. Preparation of PSO- and 5-MOP-HP-β-CD Inclusion Complex

The inclusion complexes of PSO, 5-MOP, and HP-β-CD were prepared by the freeze-drying method at a 1:1 molar ratio based on the results of phase solubility studies. PSO and 5-MOP were dissolved in 10 mL of HP-β-CD aqueous solution and individually shaken in borosilicate tubes (150 rpm, 37 °C, 7 d). Samples were centrifuged (4000 rpm, for 5 min) and filtered (Nylon filter; 0.45 µm) to remove any free drug. The solutions were then frozen and lyophilized (−20 °C, 905 mTOR) to obtain the inclusion complexes.

### 3.4. Physical Mixture (PM)

A physical mixture of PSO or 5-MOP and HP-β-CD (molar ratio of 1:1) was formed by mixing both pulverized powder components in a suitable flask, for about 10 min, until a uniform mixture was obtained and then passed through sieve (#100). The obtained products were stored in a desiccator at room temperature. 

### 3.5. Characterization of the PSO-and 5-MOP-HP-β-CD Inclusion Complex in Solid State

Inclusion complex of PSO- and 5-MOP-HP-β-CD was investigated in solid state by means of Fourier transform infrared spectroscopy (FTIR) with an attenuated total internal reflection (ATR), differential scanning calorimetry (DSC), thermogravimetric analysis (TGA), and scanning electron microscopy (SEM).

#### 3.5.1. Differential Scanning Calorimetry (DSC)

The DSC curves of PSO, 5-MOP, HP-β-CD, PSO-HP-β-CD, or 5-MOP-HP-β-CD inclusion complex (1:1) and physical mixtures were obtained using a DSC-60^®^ calorimeter (Shimadzu, Kyoto, Japan) operated with a nitrogen atmosphere at 50 mL.min^−1^, heating rate of 10 °C·min^−1^, at the temperature range of 25–300 °C. Samples of about 1.0 ± 0.5 mg were sealed into closed aluminum crucible for the analyses.

#### 3.5.2. Thermogravimetry (TG)

TG analyses were performed on a TGA/DTA-60^®^ thermobalance (Shimadzu, Kyoto, Japan) under nitrogen atmosphere (flow rate of 50 mL min^−1^). Samples (5.0 ± 1.0 mg) were placed in platinum crucibles and heated at a rate of 10 °C min^−1^, from 25 to 500 °C. All analyses were performed on PSO, 5-MOP, HP-β-CD, PSO-HP-β-CD, or 5-MOP-HP-β-CD inclusion complex (1:1) and physical mixtures.

#### 3.5.3. Fourier Transform Infrared Spectroscopy (FTIR)

FTIR spectra were obtained with a Varian 640-IR FTIR^®^ spectrometer (Varian Inc., Palo Alto, CA, USA), equipped with a universal ATR sampling accessory. A small quantity of the individual compounds (PSO, 5-MOP, and HP-β-CD) or PSO-HP-β-CD and 5-MOP-HP-β-CD inclusion complex (1:1) and physical mixtures were placed on the diamond ATR crystal. Analysis was performed over a wavelength ranging from 4000 to 400 cm^−1^ with a resolution of 4 cm^−1^.

#### 3.5.4. Scanning Electron Microscopy (SEM)

Morphology of PSO, 5-MOP, HP-β-CD, PSO-, and 5-MOP-HP-β-CD inclusion complex were determined using scanning electron microscopy (SEM). All samples were fixed in stubs using double-sided carbon tape and coated with a thin layer of gold by a sputter-coated unit (Denton Vacuum Desk V, Moorestown, NJ, USA) to make them electrically conductive prior to imaging. In this experiment, a JEOL JSM-6610 (Thermo Scientific, Madison, WI, USA) scanning electron microscope was used. The surface topography was analyzed with a scanning electron microscope operated at an acceleration voltage of 5 kV, and obtained micrographs were examined at ×100, ×500, and ×1000.

### 3.6. Phase Solubility of the PSO- and 5-MOP-HP-β-CD Inclusion Complex

Phase solubility studies were performed in KRB buffer solutions (pH 7.4), as described by Higuchi and Connors [47]. Excess amounts of PSO (2.5 mg), 5-MOP (2.5 mg), and EBGT (45.0 mg) were individually shaken in borosilicate tubes (150 rpm, 37 °C, 7 d), containing HP-β-CD of different concentrations (2.5–70.0 mM; 5 mL). Samples were centrifuged (4000 rpm, 5 min) and filtered (Nylon filter; 0.45 µm) prior to the psoralens content analysis by the HPLC-DAD validated method. The K_c_ (stability constant) was calculated based on the phase solubility diagram according to equation:

K_c_ = slope/S_0_ (1 − slope) (1)

where S_0_ is the intrinsic solubility of PSO and 5-MOP (in the absence of HP-β-CD).

The complexation efficiency (CE) was determined by using the slope of the linear phase solubility diagram as follows:

CE = slope/(1 − slope)(2)

### 3.7. Nuclear Magnetic Resonance Characterization (NMR)

NMR experiments were performed at 25 °C on a Bruker Avance III 500 spectrometer, operating at 11.75 T, observing ^1^H at 500.13 MHz, and using D_2_O as solvent. The spectrometer was equipped with a 5 mm triple inverse detection three-channel (^1^H, ^2^H, and X-nucleus—BBI) probe, tuned and matched for each sample analysis. ^1^H NMR experiments were acquired using a single excitation pulse sequence (*zg* Bruker), 8 scans with an acquisition time of 2.62 s, 64 k time domain points distributed in a spectral width of 25 ppm, and recycle delay of 1 s. For each sample analyzed, shimming was adjusted for a 90° calibrated pulse length. The results were analyzed using TopSpin and GNAT software. ^1^H and ^13^C chemical shifts are given in *δ* (ppm), related to a sodium 3-trimethylsilyl-propionate-2,2,3,3-d_4_ (TMSP) signal at *δ* 0.00 as an internal reference. 

^1^H NMR and Nuclear Overhauser Effect (NOESY) experiments were carried out with the same solutions from PSO and 5-MOP in HP-β-CD (15 mM) used in the phase solubility studies, then dissolved in D_2_O. Samples were transferred to 5 mm NMR tubes, and each signal of the furanocoumarins observed in ^1^H NMR spectra was irradiated in a NOE experiment (*selnogp* pulse sequence, Bruker, Billerica, MA, USA). Selective refocusing with a shaped pulse was used in the NOE experiments [48], with a mixing time of 500 ms and a relaxation delay of 2 s. 

^1^H diffusion measurements (DOSY—Diffusion Ordered Spectroscopy) were carried out, applying a stimulated-echo NMR pulse sequence (*stebpgp1s*, Bruker) and delay of 0.2 and 0.6 ms, respectively, for gradient recovery (d16) and duration of the gradient purge pulse (p19). The total diffusion-encoding pulse duration (p30) and diffusion delay Δ (d20) were, respectively, 2 ms and 100 ms. These values provided an attenuation of ~99% in the water signal with the maximum gradient strength. A total of 32 nominal gradient amplitudes ranging from 5 to 95% of the gradient magnitude were chosen to give equal steps in the gradient squared. The ^1^H DOSY spectra were constructed with 32 k data points in the GNAT processing with one zero filling and line broadening factor of 1 Hz.

All prepared samples (PSO and 5-MOP in HP-β-CD 15 mM) had a high amount of KRB buffer and lower concentration of psoralens, resulting in low-intensity ^1^H signals and difficulties to tune the probe. Thus, new samples without KRB were prepared to assess the diffusion of the furanocoumarins by DOSY as follows: 2.50 mg of PSO + 303 mg de HP-β-CD + 550 µL de D_2_O; 2.62 mg de 5-MOP + 238 mg de HP-β-CD + 500 µL de D_2_O; 210 mg of HP-β-CD + 450 μL de D_2_O (blank).

### 3.8. Ex Vivo Permeability

Study protocol for female Wistar rats (12–14 weeks old) was approved by the Ethics Committee on Animal Use (#066/15) at the Federal University of Goiás, Goiás, Brazil. One week before starting the experiment, all animals were acclimatized at controlled conditions with regulated access to a standard food (22 ± 2 °C, 12-h light/dark cycle). After this, they fasted for 12 h with free access to water. All animals (body weight = 215 ± 12 g) were then anesthetized using a mixture of ketamine/xylazine (90.0/7.5 mg/kg). Rat jejunum was removed and immediately placed into an ice-cold KRB solution. The jejunum (20 mm) seromuscular layer was carefully stripped off from the membrane and mounted in the MTS-Snapwell system [39]. Next, unidirectional drug transport was measured from the apical to basolateral side (A–B).

Solutions from PSO, 5-MOP, and EBGT in HP-β-CD (2.5–70.0 mM) were prepared in the physiological media, i.e., KRB (pH 7.4), as described in phase solubility studies. Sample aliquots (400 µL) were placed in the donor compartment (apical side). 

Drug transport was measured by collecting samples (500 µL) from the receiver chamber (basolateral side) at 0, 20, 30, 40, 60, 90, and 120 min. Receiver samples were replaced with equal volume of fresh KRB solution (37 °C). The system was maintained on a plate shaker (60 rpm) inside a humidified incubator (37 ± 0.5 °C) in a controlled atmosphere (95% O_2_, and 5% CO_2_), before and during the transport experiment. Samples were analyzed by the HPLC-DAD validated method. 

Transport experiments were carried out under sink conditions, i.e., the concentration in the receiver chamber did not exceed 10% of the concentration in the donor chamber [49]. The change in donor and receiver concentrations was taken into consideration when the apparent permeability coefficient (P_app_) was calculated using to the following equation:

P_app_ = dQ/dt(1/C_D_ A) (3)

where dQ/dt is the appearance rate of the drug in the receiver compartment, C_D_ is the concentration of the drug in the donor chamber at each sample interval, and A is the surface area (0.1 cm^2^) of the tissue segment. The initial concentration of the drug in the donor compartment was determined experimentally, and donor concentrations at each time point were obtained by the calculation of the mass balance [49,50].

Integrity of intestinal tissue segments mounted in the MTS-Snapwell system was assessed by measuring the transepithelial electrical resistance (TEER) using the KRB solution at 37 °C as the conducting medium. Viable intestinal segments (>30 Ω·cm^2^) were mounted on the permeation apparatus and subjected to a preincubation period (30 min) under a controlled atmosphere (95% O_2_ and 5% CO_2_) before starting the experiments.

### 3.9. HPLC-DAD Method

Analysis of EBGT markers was carried out according to a previously validated method [2]. Briefly, chromatographic analysis was performed using an HPLC Agilent 1260 system (Agilent Technologies, Santa Clara, CA, USA) equipped with a photodiode array (DAD) and a fluorescence detector (FLD). The mobile phase consisted of acetonitrile as solvent A and 0.04% trifluoroacetic acid as solvent B. PSO and 5-MOP were assayed by the HPLC-DAD (Infinity 1260, Agilent Technologies) on a Phenyl-hexyl column (150 × 4.6 mm, 5 µm, Phenomenex) using 40% A for 7.0 min at a flow rate of 1.0 mL min^−1^ and detection at 245 nm. Sample injection volume was 25 µL.

### 3.10. Statistical Analysis

GraphPad Prism 5.0 and Statistica 7 softwares were used for statistical analysis. Results are expressed as means ± standard deviation (SD). Phase solubility studies were run in triplicate (*n* = 3), and P_app_ experiments were run in sextuplicate (*n* = 6). Data analysis was evaluated using a Student’s *t*-test or a one-way analysis of variance (ANOVA), followed by Tukey’s multiple comparison tests (α = 0.05). Differences were scored as significant if *p* < 0.05. Thermal analyses and Fourier transform infrared spectroscopy FTIR figures were obtained by means of Origin 8 software (OriginLab Corporation, Northampton, MA, USA).

## 4. Conclusions

The complex formation of PSO and 5-MOP with HP-B-CD was shown by FTIR-ATR, DSC, TGA, NMR, and SEM analysis in both aqueous solution and solid state. Moreover, phase solubility studies indicated 1:1 inclusion complex formation with small stability constants, particularly for EBGT markers, which could explain the fact that P_app_ was less affected in plant extract than in pure compounds.

HP-β-CD was shown to be a potent aqueous solubility enhancer for PSO and 5-MOP, exerting only a mild negative effect on ex vivo jejunal permeability. PSO and 5-MOP solubility were improved (up to 10- and 31-fold, respectively) upon the addition of 70 mM HP-β-CD to aqueous media while reducing permeability for psoralens and EBGT markers around to 6- to 4-fold, respectively, so that there remained at least a 2-fold improvement in the potential absorption. Therefore, using HP-β-CD as a host agent for PSO, 5-MOP, or EBGT inclusion complexes may result in similar drug plasma profiles at lower doses, decreasing possible collateral effects and toxicity. Thus, it could be a useful approach when designing future pharmaceutical formulation for the treatment of vitiligo. However, HP-β-CD amount should be carefully selected to ensure an optimum solubility and permeability balance, aiming at optimal drug bioavailability and intended therapeutic activity.

## Data Availability

Not applicable.

## Sample Availability

Samples of the compounds are not available from the authors.

## Abbreviations

EBGT, extract of *Brosimum gaudichaudii* Trécul; PSO, psoralen; 5-MOP, 5-methoxypsoralen; BCS, Biopharmaceutics Classification System; P_app_, apparent permeability coefficient; HPLC, high performance liquid chromatography; DAD, diode-array detection; KRB, Krebs-Ringer Bicarbonate buffers; TEER, transepithelial electrical resistance; CD, cyclodextrin;HP-β-CD, hydroxypropyl-β-cyclodextrin; PM, physical mixture; FTIR, Fourier transform infrared spectroscopy; DSC, differential scanning calorimetry; TGA, thermogravimetric analysis; SEM, scanning electron microscopy; NMR, nuclear magnetic resonance; KC, stability constant; CE, complexation efficiency; UWL, Unstirred water layer; AUC, Area under curve; GIT, Gastrointestinal tract.
